# Application of the Mike21C model to simulate flow in the lower Mekong river basin

**DOI:** 10.1186/s40064-016-3637-8

**Published:** 2016-11-15

**Authors:** Truong An Dang, Tuan Hoang Tran

**Affiliations:** 1 Sustainable Management of Natural Resources and Environment Research Group, Faculty of Environment and Labour Safety, Ton Duc Thang University, 19 Nguyen Huu Tho Str., Dist 7, Ho Chi Minh City, Vietnam; 2 Sub-Institute of Hydro-meteorology and Climate Change, 19 Nguyen Thi Minh Khai St., Dist. 1, Ho Chi Minh City, Vietnam

**Keywords:** Numerical model, Bank erosion, Suspended load, Bed level variation, Curvilinear grid

## Abstract

Numerical models are useful tools that play an important role in many research projects. Mike21C is one of the most well-established models for simulating a variety of processes, including bank erosion, bed level variations, aggradation, and degradation. Such processes are caused by a variety of activities, such as construction and dredging, as well as seasonal flow fluctuations. Mike21C based on an orthogonal curvilinear grid, which enables a computational speed that is faster than that of other grids. The hydrodynamic part of the model is based on solving the Saint-Venant equations. In this research, the Mike21C model was applied to simulate water depth, flow discharge distribution, and suspended transport rate along reaches of the Bassac River the on Vietnam. The data files of the curvilinear grid and bathymetry were used to generate the model. A time series for the discharge and water level of hydrological stations was established. The model was calibrated using the water level and suspended load data collected during high and low flow discharges. The simulation results show that the model can be applied well to other areas.

## Background

The river watershed herein, which is one of the two large branches of the Mekong River basin, flows towards the Cuu Long River Delta. Annually, in the flood season, the rice fields receive rich alluvial and abundant fresh water from the upper catchment of the Mekong River. Despite these advantages, this river reach still suffers many unsolved problems, such as floods, salt intrusion, shortage of water in the dry seasons, and river bank erosion. The Hau River, which flows through Long Xuyen City in An Giang province is one of the two large branches of the lower Mekong River basin. In recent years, the hydrodynamic processes in the river reach have been changing in a rapid and complex manner. In particular, the river bank erosion has become a very serious issue along both sides of this curved river reach, which also has many branches flowing around the by many islands. The river reach has many holes and deep alluvial ground water, along with both sides. Two sides of the study river reach are exhibiting bank erosion phenomena (Dac 1986; Lai 2010; Tram 1985).

## Mike21C model description

In this paper, the Mike21C model is used to simulate the water depth, flow discharge, suspended load and bed load concentration of the study river reaches in the flood and dry season. Mike21C is one of the most comprehensive and well-established tools for simulating river bed and channel planform development caused by changes in the hydraulic regime. Simulated processes include alluvial resistance, bank erosion, and scouring and shoaling caused by various activities, such as construction and dredging, and seasonal flow fluctuations (Khue 1985; Lai 1987; Jin and Steffler^1993^). This model is approximated by using FDM in curved coordinates (Ahmadi et al. 2009; Beck and Basson 2007; DHI 1995; Dang and Park 2016; Talmon 1992; Gulkac 2005; McGuirk and Rodi 1978). Structurally, Mike21C has three main modules: the flow module, sediment transport module, and river morphology module. Mike21C model is applied to simulate the water level fluctuation, flow discharge distribution, suspended load transport rate, and bed level variation in the river downstream.

### Hydrodynamic module

The flow module based on the three-dimensional (3D) hydrodynamic model is complex. Application of the 3D model for simulating long time scales (e.g., months, season, and years) elevation to river morphology is a complicated process. To overcome this obstacle, scientists have converted the main hydrodynamics module into 2D equations representing the conservation of momentum and mass horizontally (DHI 1995; Ye and McCorquodale 1997).

The hydrodynamic equations are expressed as follows:

1 $$\frac{{\partial {\text{p}}}}{{\partial {\text{t}}}} + \frac{\partial }{{\partial {\text{s}}}}\left( {\frac{{{\text{p}}^{ 2} }}{\text{h}}} \right) + \frac{\partial }{{\partial {\text{n}}}}\left( {\frac{\text{pq}}{\text{h}}} \right) - 2\frac{\text{pq}}{{{\text{hR}}_{\text{n}} }} + \frac{{{\text{p}}^{ 2} - {\text{q}}^{ 2} }}{{{\text{hR}}_{\text{s}} }} + {\text{gh}}\frac{{\partial \,{\text{H}}}}{{\partial {\text{s}}}} + \frac{\text{g}}{{{\text{C}}^{ 2} }}\frac{{{\text{p}}\sqrt {{\text{p}}^{ 2} + {\text{q}}^{ 2} } }}{{{\text{h}}^{ 2} }} = {\text{RHS}}$$

2 $$\frac{{\partial {\text{q}}}}{{\partial {\text{t}}}} + \frac{\partial }{{\partial {\text{s}}}}\left( {\frac{\text{pq}}{\text{h}}} \right) + \frac{\partial }{{\partial {\text{n}}}}\left( {\frac{{{\text{q}}^{ 2} }}{\text{h}}} \right) + 2\frac{\text{pq}}{{{\text{hR}}_{\text{s}} }} + \frac{{{\text{q}}^{ 2} - {\text{p}}^{ 2} }}{{{\text{hR}}_{\text{n}} }} + {\text{gh}}\frac{{\partial {\text{H}}}}{{\partial {\text{n}}}} + \frac{\text{g}}{{{\text{C}}^{ 2} }}\frac{{{\text{q}}\sqrt {{\text{p}}^{ 2} + {\text{q}}^{ 2} } }}{{{\text{h}}^{ 2} }} = {\text{RHS}}$$

3 $$\frac{{\partial {\text{H}}}}{{\partial {\text{t}}}} + \frac{{\partial {\text{p}}}}{{\partial {\text{s}}}} + \frac{{\partial {\text{q}}}}{{\partial {\text{n}}}} - \frac{\text{q}}{{{\text{R}}_{\text{s}} }} + \frac{\text{p}}{{{\text{R}}_{\text{n}} }} = 0$$

where s, n are the co-ordinates in the curvilinear co-ordinate system; h is the water depth; p and q are the mass fluxes in the s-and n directions, respectively. H is the water level; C is the Chezy roughness coefficient; g is the gravitational acceleration; R_s_ and R_n_ are the radius of curvatures of s- and n-line, respectively; RHS is the right hand side describing the Reynolds’ stresses.

### Sediment transport module

In the sediment transport module, the suspended load transport equations under the control of convection and diffusion are expressed as follows (Duc 2004; Meyer and Müller 1948; Galappatti 1983; Jia and Wang 2001):

4 $$\frac{{\partial {\text{c}}}}{{\partial {\text{t}}}} + {\text{u}}\frac{{\partial {\text{c}}}}{{\partial {\text{x}}}} + \nu \frac{{\partial {\text{c}}}}{{\partial {\text{y}}}} + {\text{w}}\frac{{\partial {\text{c}}}}{{\partial {\text{z}}}} = {\text{w}}_{\text{s}} \frac{{\partial {\text{c}}}}{{\partial {\text{z}}}} + \frac{\partial }{{\partial {\text{x}}}}\left( {\varepsilon \frac{{\partial {\text{c}}}}{{\partial {\text{x}}}}} \right) + \frac{\partial }{{\partial {\text{y}}}}\left( {\varepsilon \frac{{\partial {\text{c}}}}{{\partial {\text{y}}}}} \right) + \frac{\partial }{{\partial {\text{z}}}}\left( {\varepsilon \frac{{\partial {\text{c}}}}{{\partial {\text{z}}}}} \right)$$

where z is the vertical coordinate; W_S_ is the settling velocity of the sediment particles, c is the suspended load concentration; ε is the eddy viscosity coefficient; u, v, and w are the flow velocity components in the x-, y-, and z-directions, respectively.

Ignoring the limited diffusion outside of the vertical diffusion, (4) becomes:

5 $$\frac{{\partial {\text{c}}}}{{\partial {\text{t}}}} + {\text{u}}\frac{{\partial {\text{c}}}}{{\partial {\text{s}}}} + {\text{v}}\frac{{\partial {\text{c}}}}{{\partial {\text{n}}}} + {\text{w}}\frac{{\partial {\text{c}}}}{{\partial {\text{z}}}} = {\text{w}}_{\text{s}} \frac{{\partial {\text{c}}}}{{\partial {\text{z}}}} + \frac{\partial }{{\partial {\text{z}}}}\left( {\varepsilon \frac{{\partial {\text{c}}}}{{\partial {\text{z}}}}} \right)$$

Bed load (S_bl_) is very closely related to the suspended load. Many formulas of the bed load transport are based on the calibration coefficients k_b_ and k_s_. Engelund and Hansen (1967) had established the relationship k_b_ + k_s_ = 1, which is used in many models, including Mike21C (DHI 1995).

6 $${\text{S}}_{\text{bl}} \, = {\text{ k}}_{\text{b}} \cdot {\text{S}}_{\text{tl}}$$

7 $${\text{S}}_{\text{sl}} \, = {\text{ k}}_{\text{s}} \cdot {\text{S}}_{\text{tl}}$$

where k_s_ and k_b_ are the suspended load and the bed load coefficients, respectively

S_tl_ is the total volume of sediment transported determined according to the formula:

8 $${\text{S}}_{\text{tl}} = 0.05\frac{{{\text{C}}^{2} }}{\text{g}}\uptheta^{{\frac{5}{2}}} \sqrt {\left( {\rho_{\text{ds}} - 1} \right){\text{gd}}_{50}^{3} }$$

where ρ_ds_ is the relative proportion of sediment (relative density of the sediment); d_50_ is the diameter of the sediment particles; Shields parameter θ is determined by;

9 $$\uptheta = \frac{\tau }{{\rho {\text{g(}}\rho_{\text{ds}} - 1 ) {\text{d}}_{ 5 0} }}$$

where τ is the flow shear stress; ρ is the water density (density of water); Shear flow is divided into two types: form drag and friction, as estimated based on local flow velocity u and the local Chézy C.

### Morphological module

In the river morphology module, the hydrodynamic solution must first be obtained before solving the sediment transport equation. Next, the river bed and hydrodynamic model are applied (Vriend and Struiksma 1983; Koch 1980; Mosselman 1992; Odgaard 1983; Olesen 1987; DHI 1995).

The equation describing bank erosion is given as follows:

10 $${\text{E}}_{\text{b}} = - \alpha \frac{{\partial {\text{z}}}}{{\partial {\text{t}}}} + \upbeta \frac{\text{S}}{\text{h}} + \upgamma$$

where E_b_ is the bank erosion rate in m/s; S is the near bank sediment transport; z is the local bed level; α, β, and γ are the calibration coefficients specified in the model.

**Fig. 1:**
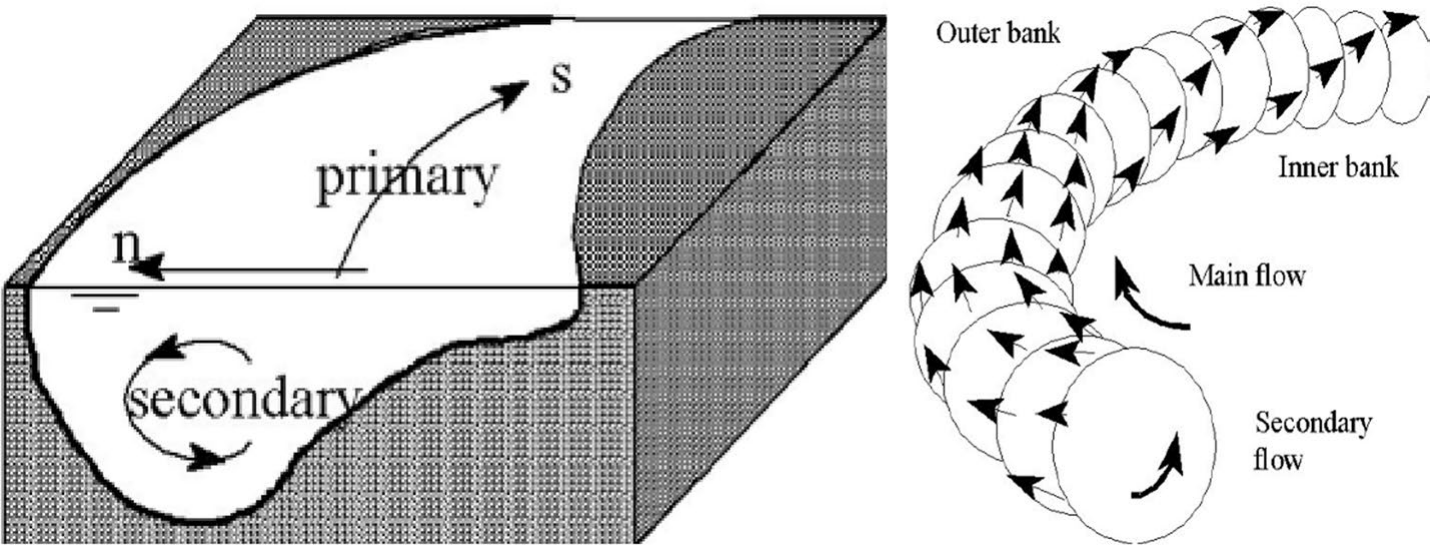
Illustration of the helicoidal flow in the river bend (DHI 1995)

Calculation of the bed level variation is based on the sediment continuity equation in the Cartesian coordinate system (Vanoni 1975; Galappatti 1983; DHI 1995):

11 $$\left( {1 - {\rm p}} \right)\frac{\partial z}{\partial t} + \frac{{\partial {\rm S}_{x} }}{\partial x} + \frac{{\partial {\rm S}_{y} }}{\partial y} = \Delta {\rm S}_{e}$$

where S_x_, S_y_ are the total volume of sediment transported along x and y, respectively; p is the porosity of the bed; ∆S_e_ is the excess sediment supply from erosion of the bed (Fig. 1).

## Mike21C application

### Study area

**Fig. 2:**
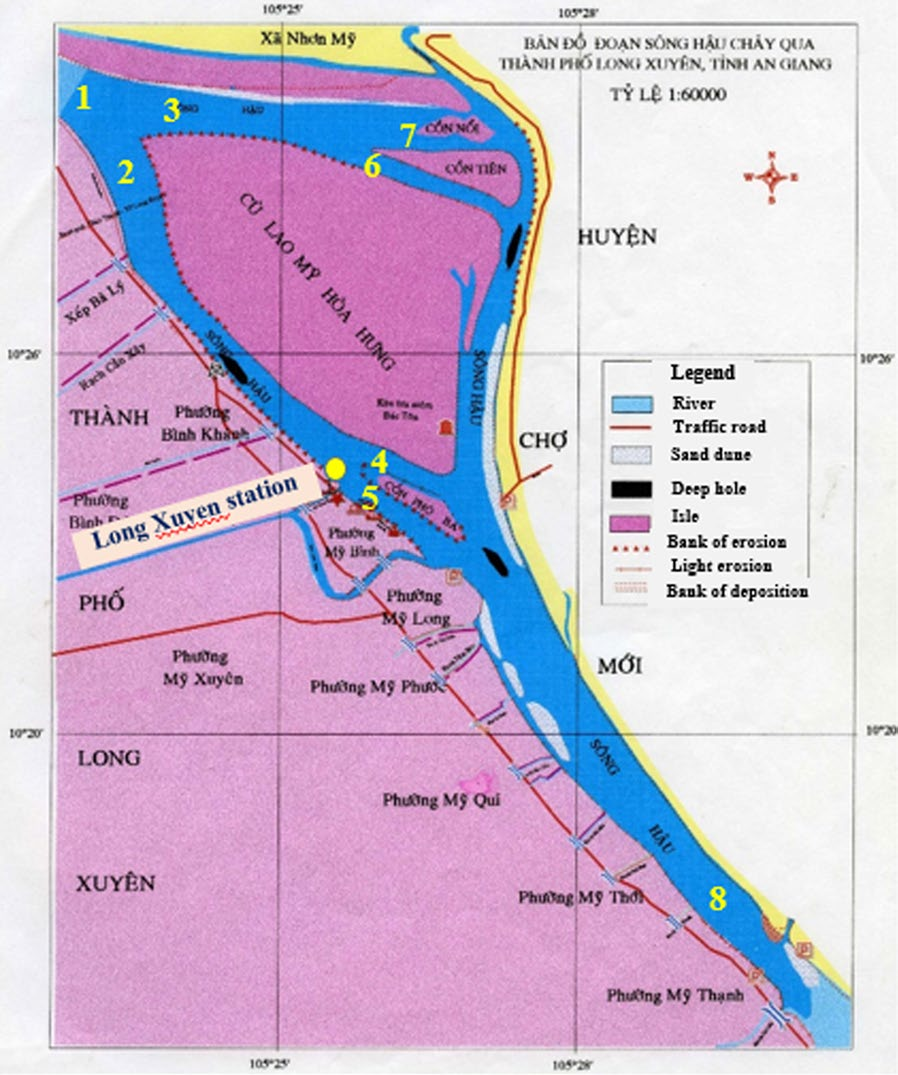
Topographic map of the study area

In this study, the Mike21C model is applied to the Hau River section belonging to the lower Mekong River Delta. The river reaches under study has a length of 20 km (Fig. 2) from the area adjacent to the Chau Thanh District downstream to the area adjacent to Can Tho City. The left side is Cho Moi District, and the right side is Long Xuyen City in An Giang province. The flood discharge in the main river at the right bank can reach up to 13,000 m^3^/s.

### Model set up

#### Initial conditions

**Fig. 3:**
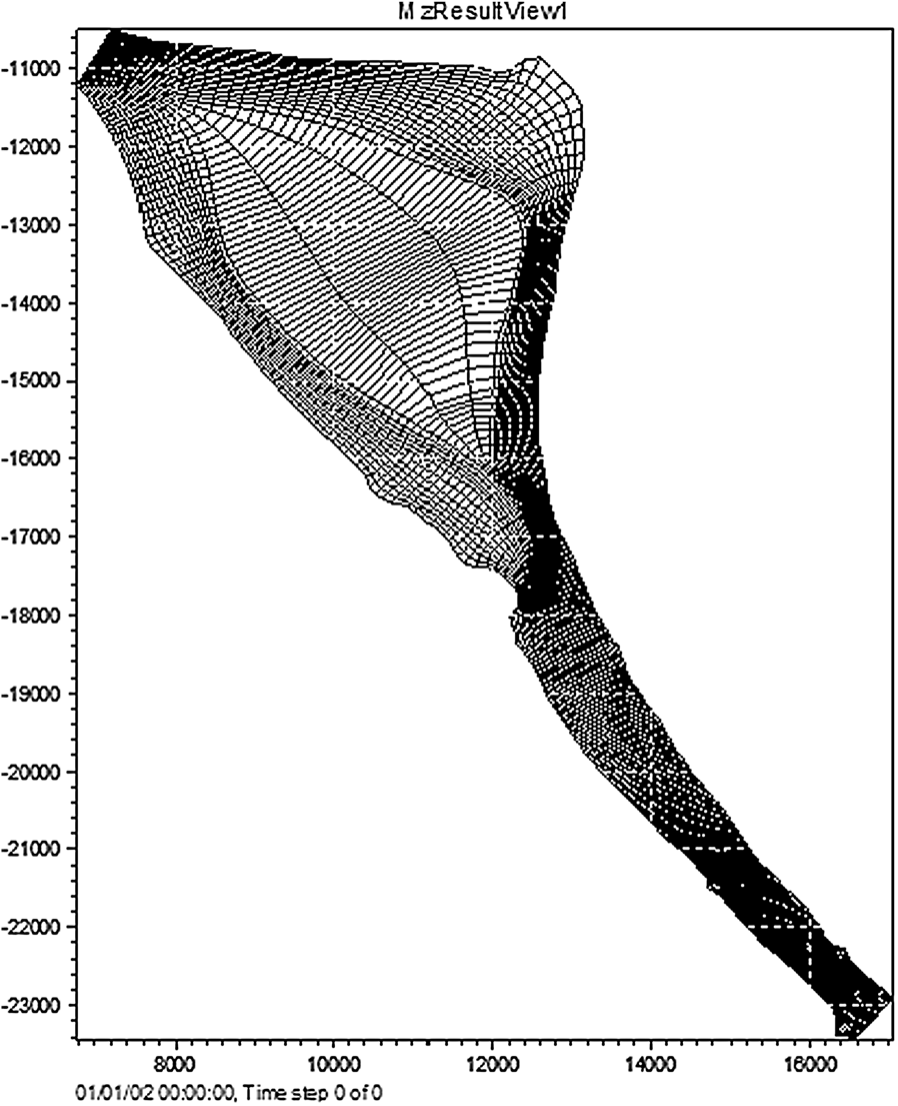
Illustration of the curvilinear grid

The important steps in the procedure to set up and solve the Mike21C model are described as follows. The first step involves the creation of a suitable curvilinear grid. A curvilinear grid is created by establishing K = 50 m, J = 100 m, and Jn × Km = 157 × 32 cells within the computational domain (Fig. 3).

**Fig. 4:**
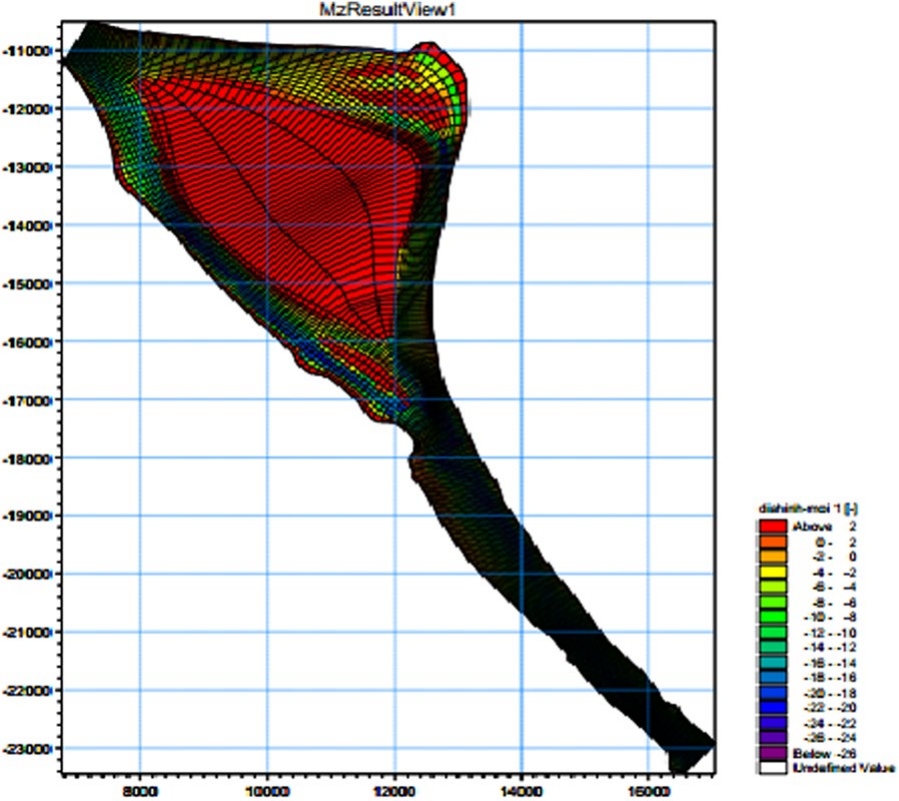
Hydraulic grid of the study river reach

Next, a bathymetry data file with coordinates and river bed level were obtained from Echo-sounding in Jan 2014. Topographic and bathymetric data measured and presented in the form of x, y, and z points, corresponding to the longitude, latitude, and water depth of the computational domain. Next, these data are imported into an excel file and interpolated into the mesh points. These mesh points are based on the original data and the interpolated mesh elevations. Detailed information on the bed topography at the initial state is given in Fig. 4.

By analyzing the mean water level for many years at the study area we found that if we select respectively the water level values of 40 cm and 160 cm and flow discharge is 3000 m^3^/s and 6500 m^3^/s in the dry season and flood season. The simulation run time would significantly reduce, because these water level values are very close to the real values of the water level.

In the numerical simulation of the open channel with irregular geometry, the water edges change with time are considered with part of the nodes being possibly wet or dry. In shallow water regions, where the water depth has a small value, the momentum terms are often ignored. The Mike21C model requires the smallest water depth to define a wet or dry cell. This dry value is set along the boundaries (Dac 1986; DHI 1995; Dang and Park 2016; Wu 2004). Manning’s coefficient and other parameters are selected as model calibration parameters along the river reach during simulation (Table 1).

**Table 1:** The main hydraulic parameters of the model

Parameters	Values	Notes
E	0.3 m^2^/s	Eddy viscosity coefficient
n	0.025	Manning’s coefficient
µ	0.7	Dynamic coefficient of friction
υ	10^−6^ m^2^/s	Kinematic viscosity coefficient
S_h_	0.7	River morphology coefficient
φ	0.2 m	Drying depth
d_50_	0.035 mm	Median grain diameter of D_50_
d_90_	1.23 mm	Median grain diameter of D_90_
p	0.4	Porosity

#### Boundary conditions

The inflow and outflow boundaries that describe the hourly water level time series were obtained from the hydrology station of Long Xuyen (Fig. 2). The inflow boundary that describes the hourly discharge time series was obtained from the hydrology station of Long Xuyen using Acoustic Doppler Current Profiler (ADCP) measuring device. The calibration process was performed for the time period from 04 to 30 Apr 2014 (dry period) and 10 to 30 Sep 2014 (high flood period).

## Simulation results

### Flow velocity during dry season and during flood season at the study area

The calculated results of the flow velocity distribution during the driest season from the Mike21C model (Table 2) showed that the flow velocities and ebb velocities at the two ends of the river reaches are the same. The flow velocity at the head and end of the river reach is 1.27 and 1.26 m/s, respectively. The ebb velocity at the head and end of the river reach is 1.03 and 1.01 m/s, respectively. At the branch of My Hoa Hung and Pho Ba, the islet flow and ebb velocities are 1.0 m/s. At the right-hand side of the My Hoa Hung islet, the flow velocities are higher than 1.2 m/s. General simulation results are consistent with the real condition because the bed river topography in this river reach is narrow; thus, the velocities must increase.

**Table 2:** Flow velocity in dry season at the study area

No.	Section	V_downstream_ (m/s)	V_upstream_ (m/s)
1	Head of river branch	1.27	1.03
2	The right branch of My Hoa Hung islet	1.13	1.12
3	The left branch of My Hoa Hung islet	0.64	0.51
4	Head of left branch of Pho Ba islet	0.45	0.30
5	Head of right branch of Pho Ba islet	1.17	0.95
6	The right branch of Tien islet	0.62	0.41
7	The left branch of Tien islet	0.52	0.42
8	At the end of river branch	1.26	1.01

At the left-hand side of the Pho Ba, My Hoa Hung islet, Tien, Noi sand dune and at the right-hand side of islet Pho Ba and Tien sand dune, the velocities ebb and flow are smaller than 1.0 m/s due to the sediment deposition at the river bed.

According to the simulated results of the flow velocity at the flood season (Table 3) at the head of the left-hand islet of Pho Ba, My Hoa Hung, and Tien, the velocities are less than 2.0 m/s. There is only flow water and no ebb water in such seasons. At the head of the river reach, the head of the right-hand islet of My Hoa Hung, and the left-hand islet of My Hoa Hung, the velocities are over 2.0 m/s. Flow velocities greater than 2.0 m/s is the cause of frequent flooding or river bank landslides during flood season.

**Table 3:** Flow velocity in flood season at the study area

No.	Section	V_downstream_ (m/s)	V_upstream_ (m/s)
1	Head of the river branch	2.59	–
2	The right branch of My Hoa Hung islet	2.21	–
3	The left branch of My Hoa Hung islet	1.30	–
4	Head of the left branch of Pho Ba islet	1.30	–
5	Head of the right branch of Pho Ba islet	2.35	–
6	The right branch of Tien islet	1.70	–
7	The left branch of Tien islet	1.22	–
8	At the end of the river branch	1.05	–

Symbol “–” is not measured flow velocity flowing from downstream to upstream

### Flow discharge distribution

Flow discharge distribution into river branches at the driest season is Q = 6000 m^3^/s when the lowest tidal water is H = −60 cm. Table 4 shows the flow discharge in the river branches. The water discharge into the left-hand islet My Hoa Hung accounts for 84.5% of the total water discharge at the head of the river reach. At the Pho Ba islet, the water discharge is divided into 74.7% at the left-hand side of Pho Ba inlet and 9.8% at its right-hand side. Water discharge into the left-hand islet My Hoa Hung is 15.5% of the total discharge at the head of the river reach. The water discharge is divided into 8.5% at the right-hand side of the Tien sand bar and 4.6% at its left-hand side.

**Table 4:** Flow discharge distribution in dry season at the study area

No.	Section	Q_dry season_ = 6000 m^3^/s (%)
1	Head of the river branch	100
2	The right branch of My Hoa Hung islet	84.5
3	The left branch of My Hoa Hung islet	15.5
4	Head of the left branch of Pho Ba islet	9.8
5	Head of the right branch of Pho Ba islet	74.7
6	The right branch of Tien islet	8.5
7	The left branch of Tien islet	4.6
8	At the end of the river branch	2.4

Flow discharge distribution into river branches at the flood season is Q = 13,000 m^3^/s and corresponds to the highest water level of H = 200 cm. Table 5 shows the flow discharge in the river branches. Water discharge into the left-hand side of islet My Hoa Hung accounts for 83.8% and the left-hand side of the islet accounts for 16.2% of total water discharge at the head of the river. As a result, the left-hand side of islet My Hoa Hung often suffers from more serious river bank landslides compared to other regions during flood season. At the Pho Ba islet, the water discharge is divided into 71.8% at its right-hand side and 12.0% on its left-hand side. At Tien sand bar, the water discharge is divided into 8.6% at its right-hand side and 5.2% on its left-hand side.

**Table 5:** Flow discharge distribution in flood season at the study area

No.	Section	Q_flood season_ = 13,000 m^3^/s (%)
1	Head of the river branch	100
2	The right branch of My Hoa Hung islet	83.8
3	Head of the right branch of Pho Ba islet	16.2
4	Head of the left branch of Pho Ba islet	12.0
5	Head of the left branch of My Hoa Hung islet	71.8
6	The right branch of Tien islet	8.6
7	The left branch of Tien islet	5.2
8	At the end of the river branch	98.5

**Fig. 5:**
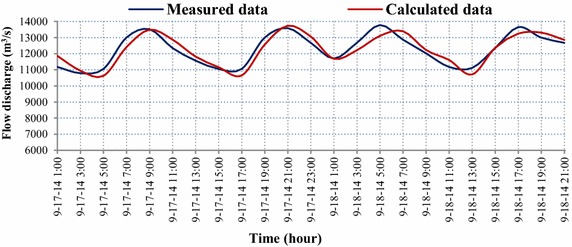
Comparison of the observed and simulated flow discharge at Long Xuyen station (see Fig. 2) on in flood season with NASH = 0.83

The simulation results of the flow discharge at Long Xuyen station 9 km away from the upstream boundary are presented in Fig. 5. In general, the simulation results of the flow discharge are in good agreement with the measured data. The difference between two successive troughs of the measured and simulated flow discharge is very small, 1.57%, whereas the difference between two successive peaks is 6.75%. By applying the Nash–Sutcliffe criterion (Nash and Sutcliffe 1970), the validation of calculated and measured flow discharge data with Nash–Sutcliffe is 0.83. This result demonstrates that the simulated model of the flow discharge is of high accuracy.

### Simulation results of the water level

**Fig. 6:**
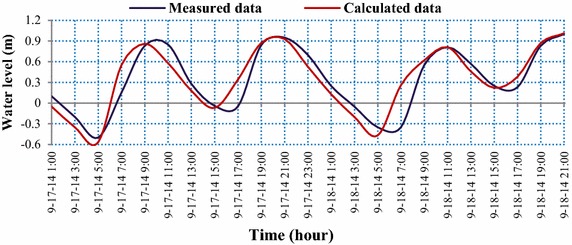
Comparison of the measured and predicted water level at Long Xuyen station (see Fig. 2) during the dry season with NASH = 0.80

The calculated results of the water level during the dry season (Fig. 6) showed that the water level oscillation at the studied river reach is semi-diurnal. The difference between two successive troughs is 20 cm, whereas the difference between two successive peaks is small. The amplitude of the flow tides (ΔH_L_) and the amplitude of the ebb tides (ΔH_X_) are the same, with a value of 110 cm on average, with the maximum value of ΔH_Xmax_ and ΔH_Lmax_ being 160 cm; the average time of the flow tide (ΔT_L_) and of the ebb tide (ΔT_X_) is 4 h 52 min and 7 h 42 min, respectively.

**Fig. 7:**
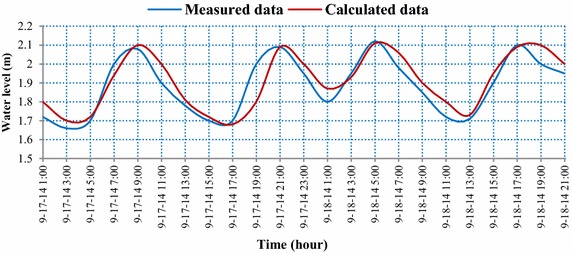
Comparison of the measured and predicted water level at Long Xuyen station (see Fig. 2) during the flood season with NASH = 0.87

Simulation results of the water level during the flood season (Fig. 7) showed that the water level oscillation at the studied river reach is semi-diurnal. The difference between two successive troughs is less than 10 cm, whereas the difference between two successive peaks is 20 cm; ΔH_Ltb_ = 23 cm, ΔH_Lmax_ = 90 cm, ΔH_Xtb_ = 27 cm, and ΔH_Xmax_ = 76 cm. The average time of flow tide ΔT_L_ and ebb tide ΔT_X_ is 5 h 35 min and 8h21 min, respectively.

The validation of the calculated and measured water level data indicates that the Nash–Sutcliffe index ranges from 0.80 to 0.87. This result implies that the simulated model of the water level is very reliable.

### Suspended load and bed load concentration distribution

The calculated results (Table 6) showed that the suspended load is low during in the dry season, approximately 0.01–0.03 kg/m^3^, and is rapidly increasing during the first heavy rains of the season, up to 0.45–0.75 kg/m^3^.

**Table 6:** Calculation results of suspended load

Branches of the study river reach	Dry season (kg/m/s)	Flood season (kg/m/s)
The right branch of the My Hoa Hung islet	0.22	18.7
The left branch of the My Hoa Hung islet	0.11	7.02

Similarly, the calculated results (Table 7) showed that the bed load is very low during the dry season and during the flood season. The bed load concentration is small, and its concentration is approximately 10–15% suspended load concentration.

**Table 7:** Calculation results of bed load

Branches of the study river reach	Dry season (kg/m/s)	Flood season (kg/m/s)
The right branch of the My Hoa Hung islet	0.02	2.06
The left branch of the My Hoa Hung islet	0.01	0.91

## Conclusions

From the simulation results, the flow velocity, water level, and sediment transport during the dry and flood seasons at the study river reach are summarized as follows:

The simulation results of the flow discharge were found to be in agreement with the observed data. The NASH index for the model calibration was 0.83. Generally, the flow discharge is small during the dry season but high during the flood season. This result shows that the study river reach is influenced by flows from the upper Mekong River.

The simulation results of the water level phase showed that the measured data are very close to the predicted data, both during the dry season and the flood season. However, slight differences of peak and trough tides were found between the calculations and the measurements. Generally, the simulated model of the water level is very reliable, with Nash–Sutcliffe values ranging from 0.80 to 0.87.

The calculated results for the suspended load and the bed load concentration in the dry season are relatively low, however, their concentration quickly increases during the flood season. This is entirely consistent with the actual conditions of the study river reach.

The results of the calibration and validation of the water level, flow discharge, and sediment transport showed that the simulation results have high reliability.

These results provide useful scientific information to help professional agencies decide on the projects to implement for the protection of the river bank to reduce damage to people and property due to the river erosion in the study river reach.

The 2D curvilinear grid hydraulic model Mike21C has proven to be is a useful tool for achieving better resolution of the flow velocity along the solid boundaries, thereby achieving higher modeling accuracy. The Mike21C model can be successfully applied to simulate river flows and address sediment transport problems associated with river morphology structures and has strong applicability to engineering practices problems.

## References

[CR1] Ahmadi MM, Ayyoubzadeh SA, Namin MM, Samani JMV (2009). A 2D numerical depth-averaged model for unsteady flow in open channel bends. J Agric Sci Tech.

[CR2] Beck JS, Basson GR (2007). Klein River Estuary (South Africa): 2D numerical modelling of estuary breaching. Water SA.

[CR3] Dac NT (1986) Model estimates a non-stop one-way transmission of tidal and salt water intrusion on canals and rivers. Dissertation, Thuy Loi University

[CR4] Dang TA, Park SD (2016). Development and testing of 2D finite difference model in open channels. Environ Model Assess.

[CR5] Danish Hydraulic Institue (1995) Coastal Hydraulics and Oceanography, Hydrodynamic Module. Release 2.5, Users Guide and Reference Manual

[CR6] Duc BM (2004). Numerical modeling of bed deformation in laboratory channels. J Hydrau Eng ASCE.

[CR7] Engelund F, Hansen E (1967). A monograph on sediment transport in alluvial streams.

[CR8] Galappatti R (1983) A depth-integrated model for suspended transport, Report no. 83-7, comm. On hydraulics, Dept. of Civil Engineering, Delft Univ. of Technology

[CR9] Gulkac V (2005). On the finite differences schemes for the numerical solution of two-dimensional moving boundary problem. Appl Math Comput.

[CR10] Jia Y, Wang SSY (2001) CCHE2D: two-dimensional hydrodynamic and sediment transport model for unsteady open channel flows over loose bed. Technical Rep. No. NCCHE-TR-2001-1, School of Engineering, Univ. of Mississippi, Oxford

[CR11] Jin YC, Steffler PM (1993). Predicting flow in curved open channel by depth-averaged method. J Hydrau Eng.

[CR12] Khue NN (1985) Computer research and development of hydraulic on the unstable river systems of Vietnam. Dissertation, Thuy Loi University

[CR13] Koch FG (1980) Bed level computation for axis-symmetric curved channels, Rep. R567-1X/W308 part 1, Delft Hyd. Lab

[CR14] Lai NV (1987) Researching and applying mathematical models and hydrological properties humid tropical river flow. Dissertation, Thuy Loi University

[CR15] Lai YG (2010). Two-dimensional depth-averaged flow modeling with an unstructured hybrid mesh. J Hydrau Eng.

[CR16] McGuirk JJ, Rodi W (1978). A depth averaged mathematical model for near field of side discharge into open channel flow. Journal Fluid Mechanic.

[CR17] Meyer-Peter E, Müller R (1948) Formulas for bed load transport, Proceedings of 2nd Congress in IAHR, Stokholm, vol 2, paper 2

[CR18] Mosselman E (1992) Mathematical modelling of morphological processes in rivers with erodible cohesive banks, communications on hydraulic and geotechnical engineering, no. 92-3, Delft University of Technology

[CR19] Nash JE, Sutcliffe JV (1970). River flow forecasting through conceptual models part I-A discussion of principles. J Hydro.

[CR20] Odgaard A (1983). Closure to “Transverse Bed Slope in Alluvial Channel Bends” by A. Jacob Odgaard (December, 1981). J Hydraul Eng.

[CR21] Olesen KW (1987) Bed topography in shallow river bends, Delft University of Technology, Report 87-1

[CR22] Talmon AM (1992) Bed topography of river bends with suspended sediment transport, Dissertation, Delft University of Technology

[CR23] Tram BD (1985) Hydrologic regime of the valley, Long Xuyen Quadrangle. Themes of State under the program code 60-02-04 investigation of the phase II contract MD 1982–1984. Science and Technology Committee published in An Giang

[CR24] Vanoni VA (1975) Sedimentation engineering, ASCE manuals and reports on engineering practice, No. 54, New York

[CR25] Vriend HJ, Struiksma N (1983) Flow and bed deformation in river bends. Proceedings of ASCE Conference Rivers’ 83, New Orleans

[CR26] Wu W (2004). Depth-averaged two-dimensional numerical modeling of unsteady flow and nonuniform sediment transport in open channels. J Hydraul Eng.

[CR27] Ye J, McCorquodale JA (1997). Depth averaged hydrodynamic model in curvilinear collocated grid. J Hydrau Eng.

